# The Treatment of Septic Peritonitis

**Published:** 1900-05-05

**Authors:** 


### THE TREATMENT OF SEPTIC PERITONITIS.

Dr. George R. Fowler,[1] Professor of Surgery to the New York Polyclinic, and Surgeon to the Methodist Episcopal Hospital, has made a most important observation in regard to the treatment of septic peritonitis. Not only does the idea seem to be good, but when put into practice it appears to have worked out well, which is the true criterion and the proper test. Starting from certain anatomical details, showing the very different degree in which the upper and lower portions of the peritoneal cavity are connected with the rest of the lymphatic system, and going on to the clinical observation that, while the presence of septic inflammation in the upper or diaphragmatic and intestinal part of the peritoneum is extremely dangerous, a very considerable degree of septicity may exist in the pelvis without serious consequences so long as it is shut off from the general peritoneal cavity, he was led to the practice which he recommends in the treatment of septic peritonitis, namely, raising the head and trunk of the patient to such a level that the effusion in the peritoneum shall sink down into its least dangerous region within the pelvis. After performing laparotomy, and adopting the usual surgical measures for removing the cause of the inflammation, he props up the patient's head and trunk, and inserts drainage tubes into the pelvis, through which the fluid is repeatedly removed as it flows down into that region. The result seems to have been admirable. He relates nine successive cases of diffuse septic peritonitis treated by the combined methods of elevated posture and drainage, all of which resulted in recovery. He has not included among these several cases in which a spreading peritonitis existed about a focus of infection, as shown by a decided redness of the coils of intestine in the neighbourhood, and which were apparently arrested by the elevated head and trunk position, although in these also the convalescence was rapid, and the condition seemed to be more comfortable than is usual during the first few hours after operation. For the purpose of comparison, Dr. Fowler takes an equal number of cases of diffuse septic peritonitis occurring in his hospital practice which were subjected to the same measures of treatment, with the exception of the elevated head and trunk posture. Of these cases four recovered and five died. The record of nine consecutive cases of diffuse septic peritonitis terminating in recovery is, he says, extraordinary, not to say startling, and whatever doubt may still cling about the questions of eventration, or the use of peroxide of hydrogen, or the flushing of the peritoneum with large quantities of decinormal saline, he has no hesitation in speaking with confidence upon two points in the treatment of septic peritonitis, namely, the employment of the elevated head and trunk position, and drainage of the pelvic cavity by means of well-placed and protected glass drains. The angle at which the patients have been placed has varied somewhat, but the elevation of the bed from the horizontal has been generally from twelve to fifteen inches.

[1] Med, Rec., April 14.

